# Clinical study evaluating antihyperglycemic efficacy and safety of *terminalia arjuna* versus sitagliptin in Type-2 diabetes mellitus patients

**DOI:** 10.6026/9732063002001862

**Published:** 2024-12-31

**Authors:** Ved Prakash, Nidhi Goel, Kiran Rajendra Giri, Ashish Goel

**Affiliations:** 1 Department of Pharmacology, Institute of Medical Sciences, Banaras Hindu University, Varanasi, Uttar Pradesh - 221005, India; 2 Department of Pathology, Baba Kinaram Autonomous State Medical College, Chandauli, Uttar Pradesh, India; 3 Department of Physiology, Graphic Era Institute of Medical Sciences, Dehradun, India

**Keywords:** Antihyperglycemic, *terminalia arjuna*, Type-2 diabetes mellitus

## Abstract

Diabetes mellitus (DM) and its complications are devastating and our literature has mentioned different potentialities of *terminalia arjuna*, including antihyperglycemic effects, but majority researches done on animals, rarely clinical studies conducted. Our 3month study enrolling 60 DM patients analyzed bark of *T.arjuna* for its antihyperglycemic effects along with safety, group TAM: *terminalia arjuna* 1gm with metformin 500mg twice daily, group SGM: Sitagliptin 100mg once, with metformin 500mg twice daily. FBS, LFT and ADRs monitored at baseline, 4, 8 and 12 weeks, while HbA1c at baseline and 12weeks, FBS and HbA1c showed significant (<0.05) reduction in both groups, the reduction in FBS at 4, 8 & 12weeks in gpSGM vs gpTAM (FBS -29.50±8.86 vs -22.57±9.28, -46.03±9.73 vs 42.13±8.95, -62.80±9.06 vs -61.17±10.22) and in HbA1c at 12 weeks was comparable gpSGM vs gpTAM (HbA1c -0.90±0.34 vs 0.89±0.29) difference was non-significant (>0.05). On monitoring ADRs, gpSGM 2/30 had headache and 1/30 had nasopharyngitis initially during first 4weeks, which was revealed later on, while none had any side effects in gpTAM. Thus, concluding that both are equally efficacious as antihyperglycemic agent but *terminalia arjuna* has slight upper hand as is better tolerated in respect to any adverse drug reaction with comparable efficacy too.

## Background:

Diabetes mellitus is a chronic and devastating, medical condition that constitutes a major public health problem [1]. Day to day its global burden is increasing, creating the demand of effective glycaemic control and limits of monotherapy gaining wider medical importance [2]. Various complications related to diabetes mellitus are grouped into macrovascular and micro vascular categories, including cerebrovascular accidents, peripheral arterial disease, coronary heart disease, peripheral neuropathy, diabetic retinopathy and nephropathy [3]. To date, no reversal of this disease pathology has been found. Various treatment modalities presently used are lifestyle-behavioural moderation and pharmaceutical interventions, including oral anti-hyperglycaemic agents, including non-sulfonyl urea secretagogues, biguanide, DPP-IV inhibitors, alpha glycosidase inhibitors, thiazolidinedione's, GLP-1 analogues, SGLT2 inhibitors, 11β-HSD-1 inhibitors and insulin [4]. They aimed at preventing and controlling hyperglycaemia, ensuring the adequate glucose delivery to the various tissues and attempting to decrease the harm caused by hyperglycaemia [3]. Sitagliptin a popularly used DPP-4 inhibitor, inhibits the proteolytic cleavage of endogenous incretins (GLP-1 and GIP), thus increasing their concentration as well as activity, which further potentiates insulin secretion along with inhibition of glucagon secretion, leading to a reduction in serum glucose levels [5]. In a rapidly evolving world, development and degradation are occurring hand in hand, Our vast medicinal system too is curing on the one hand and causing adverse effects on the other hand, leading to this turning to the natural and the indigenous treatment methodologies, which are becoming a need of society, ancient old medicinal system-Ayurveda is filled with knowledge regarding medicinal plants and their valuable products, but it is also lacking in adverse effects [6]. In modern medicine as well, plants have been the major source of medicinal drugs for the many decades, *terminalia arjuna* belonging to the family Combretaceae, is one of the such popular medicinal plant described in ayurvedic literature [7]. Various parts of *terminalia arjuna* have medicinal values, among them the bark of the tree, which occupies pride of place [6]. Many researches have used it in powder form mainly, various biologically active chemical compounds, including tannins, triterpenoids, flavonoids and minerals such as calcium, copper, zinc and magnesium, have been found in *T.arjuna* extracts and *T.arjuna* showed no renal, hepatic or hematological adverse effects even after prolonged administration [7]. Experimental and clinical research has concluded the cardio-protective properties *terminalia arjuna* as hypolipidemic, inotropic and antioxidant [8]. Traditional healers and few experimental studies claim the antidiabetic properties possessed by its bark, several studies confirmed its antihyperglycemic, analgesic and antioxidant properties were due to flavonoids while cardio-protective activity is due to triterpenoids [9]. Over the previous years, there has been a reinvigoration of research-based studies investigating the medicinal properties of the *terminalia arjuna* mainly concluding its cardio-protective values, but few research studies have been conducted with respect to its antihyperglycemic effects that too experimental studies. Through this clinical research, we are analyzing the hypoglycaemic property of T.arjuna in comparison with Sitagliptin. Therefore, it is of interest to report the significant hypoglycemic property of *T.arjuna*.

## Materials & Methods:

*terminalia arjuna* was procured from National botanical research institute (NBRI), Lucknow and was identified and confirmed by department of Dravyaguana, state ayurvedic college and hospital, Lucknow. A well identified bark was then procured for further processing. It was washed in running tap water, then dried in shade and processed to a dry powder form. The sieved dry powder was carefully stored in airtight containers, later on was filled in capsules (500mg/capsule) at Gray's Remedies Pvt. Ltd. Ambala Cantonment, Haryana. Inclusion/exclusion criteria: Patients of either sex of age ≥ 20 years with type-2 Diabetes Mellitus having FBS ≥ 126 mg/dl, & ≤200 mg/dl HbA1c > 6.5% & < 10% were included, while any patients with heart disease, hepatic or renal failure, type-1 diabetes mellitus, DM type 2 with known complications, lactating mothers, or pregnant women were excluded. This 3-month randomized, prospective clinical study was done with institutional ethical committee permission on 60 DM type-2 patients coming to the medicine department OPD, with written informed consent. The Declaration of Helsinki was followed for all procedures required. Enrollment of patients was done on fulfillment of inclusion and exclusion criteria. Personal and past history, along with relevant clinical examination details of study patients, was recorded. Patients were informed to immediately stop the treatment if any undesirable symptoms occurred after the start of treatment and to report us immediately. Selected patients were divided randomly into 2 groups, with thirty patients in each, *i.e.*, groups TAM & SGM. gpTAM was prescribed 1gm *terminalia arjuna* twice daily, along with 500mg Metformin twice daily. gpSGM - was prescribed 100mg Sitagliptin once daily and 500mg metformin twice daily. All patients were suggested to follow lifestyle modifications, including a low-fat, high-carbohydrate diet and regular exercise. The patient's FBS level and LFT readings were recorded at the first visit, then after 4, 8 and 12 weeks, while HbA1c was recorded at the first visit and at 12 weeks of starting the treatment. Finally, the baseline values, *i.e.*, the first visit values of all parameters, were compared and analyzed with the values at 4, 8 and 12 week values, intragroup comparison using Paired 't' test and intergroup comparison by the unpaired 't' test and then evaluated for efficacy and safety profile of both the study drugs.

## Results:

## Demographic analysis:

Study gpTAM with a mean age of 51.93±7.75 years, comprised of 40% (12/30) males and 60% (18/30) females, while gpSGM has 46.7% (14/30) males and 53.3% (16/30) females with a mean age 52.27±7.45 years. The study groups were similar in respect to demographic details when statistically analyzing the data (p > 0.05) (Table 1).

## Chronicity of study disease (DM type-2):

The mean of diabetic duration in group TAM and group SGM were 3.78±1.22 years and 3.45±0.98 years, respectively which again was statistically non-significant (p > 0.05) on comparing, hence groups were comparable (Table 1).

## Past / personal history:

On statistically comparing the data for past history, which includes chronic heart disease and hypertension, as well as personal history, which emphasized on the habit of smoking, alcohol and sedentary lifestyle of the patients, the difference was non-significant (p > 0.05) (Table 1).

## Baseline analysis of study parameters:

**Figure 1:**
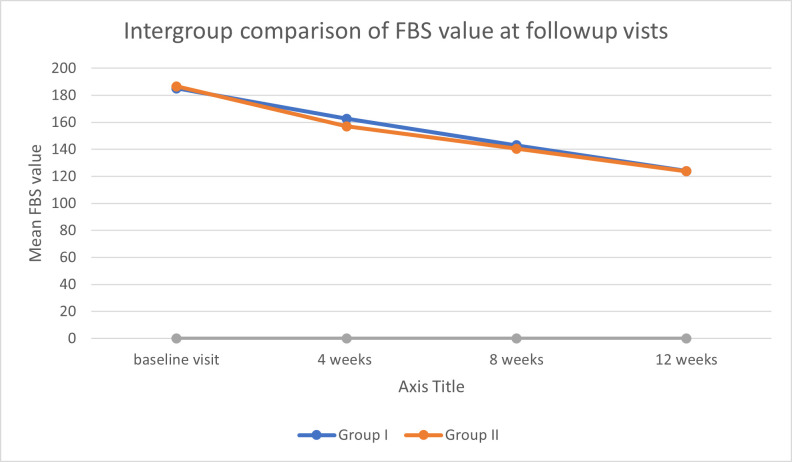
Intergroup comparison of FBS value at follow-up visits

**Figure 2:**
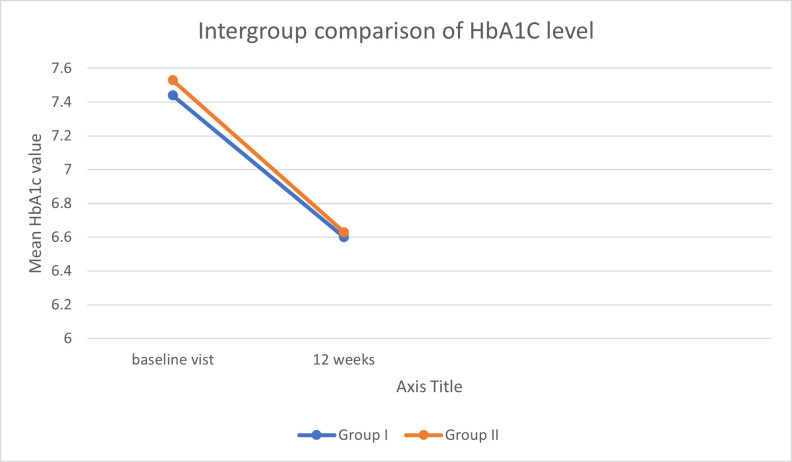
Intergroup comparison of HbA1c level

Mean values at baseline visits of gpTAM were FBS-185.20±12.16, HbA1c-7.44±0.47, SGPT-25.60±4.66, SGOT-26.30±4.02, ALP-70.07±11.15 and of gpSGM were FBS-186.60±11.67, HbA1c- 7.53±0.51, SGPT-28.70±3.84, SGOT- 29.83±4.42, ALP-75.33±8.83. On analyzing and comparing the baseline values for these parameters' variation was statistically non-significant (p>0.05) (Table 1). In gpTAM patients, fasting blood sugar level at baseline, 4-week, 8-week and 12-week visits is 185.20±20.16, 162.62±13.09, 143.07±10.33 and 124.03±7.95, respectively, showing a noticeable reduction in fasting blood sugar level, leading to mean change of 22.57±9.28, 42.13±8.95 and 61.17±10.22 at follow-up visits on comparing with the baseline value. HbA1c also showed a decrease from baseline visit (7.44±0.47) to 12-week visit (6.59±0.32), with a mean change of 0.89±0.29. The data, when analyzed statistically, was found to be highly significant (p-value<0.001) (Table 2). In gpSGM patients, fasting blood sugar level at baseline, 4-week, 8-week and 12-week visits is 186.60±11.67, 157.10±12.07, 140.57±11.19 and 123.80±7.56, respectively, showing a noticeable reduction in fasting blood sugar level, leading to a mean change of 29.50±8.86, 46.03±9.73 and 62.80±9.06 at follow-up visits on comparing with the baseline value. HbA1c also showed a decrease from the baseline visit (7.53±0.51) to the 12-week visit (6.63±0.29), with a mean change of 0.90±0.34. The data, when analyzed statistically, was highly significant (p-value<0.001) (Table 3). Intergroup comparison of gpTAM with gpSGM FBS level at baseline, follow-up 4-week, 8-week and end-point visits showed a mean change difference of -1.40, 5.53, 2.50 and 0.23 respectively while mean change difference of HbA1c at baseline and at endpoint visit is -0.09 and -0.04 respectively. The statistical analysis of the data showed the variation between the group results is non-significant (p>0.05) (Table 4) (Figure 1, Figure 2).

None of the LFT parameters, including SGPT, SGOT and ALP, showed any deterioration at any point of visit in both groups, TAM and SGM. The statistical analysis on comparing the data for both groups was also non-significant. 3/30 (10%) patients in gpSGM had minor complaints of mild adverse effects during the first 4 weeks of starting the treatment, that comprised of headache (2/30, 6.67%) and nasopharyngitis (1/30, 3.33%), while none of the patient had any complaint of side effects in gpTAM and although this difference is also non-significant on analysis (p>0.05) (Table 5).

## Discussion:

DM type 2 is among the most common 'lifestyle disorders' as its major etiological factor is day-to-day lifestyle adaptations according to fast-paced life, including dietary changes, causing imbalance between body internal mechanisms and external environment, leading to disturbance of circadian rhythms. There is no cure for this disease yet. The present available treatment targets are to maintain the blood glucose level so that adequate glucose delivery to the body tissue occurs and to avoid any damage to different organs due to hyperglycaemia or hypoglycaemia. Various synthetic drugs are available in the market that only partially compensate for the metabolic disturbances caused by diabetes, with accompanying adverse effects. With increasing prevalence of diabetes, urgent expansion of effective treatment modalities without side effects and cost-effective is today's need. The present study was conducted with the aim of confirming the hypoglycaemic effect of *terminalia arjuna*, a natural DPP-4 inhibitor, on patients diagnosed with type-2 DM, along with an assessment of its efficacy and safety by comparing with a novel synthetic DPP-4 inhibitor Sitagliptin, popularly used oral anti-hyperglycaemic drug. *terminalia arjuna* a popular medicinal herb, reported to possess inotropic, cardiotonic, antioxidant, antiallergic and hypocholesterolemic effects [10, 11, 12]. Experimental researches on rats have recently demonstrated the DPP-4 inhibitory, anti-diabetic effects of *terminalia arjuna* [13] The present study was done on sixty diagnosed cases of diabetes mellitus, randomly distributed into 2 groups, with each enrolling 30 patients. Group TAM was prescribed 1gm *terminalia arjuna* with 500mg of metformin twice daily, while group SGM was prescribed 100mg Sitagliptin once daily with 500mg of metformin twice daily. FBS, HbA1c and LFTs of all the study patients were recorded at the start of treatment, follow-up visits and endpoint for analysis of the results. The FBS level showed a noticeable reduction on every follow-up visit, with a mean change of 22.57±9.28, 42.13±8.95 and 61.17±10.22 at 4, 8 and 12 weeks from the baseline value, in patients on *terminalia arjuna* and the reduction was analyzed to be highly significant (p<0.001) on statistical analysis. This result was comparable to group SGM patients taking Sitagliptin, showing a mean change of FBS 29.50±8.86, 46.03±9.73 and 62.80±9.06 at 4, 8 and 12 weeks from baseline level and the reduction again was analyzed to be highly significant (p<0.001). Similarly, HbA1c reduction (mean change group TAM-0.89±0.29 vs. group SGM-0.90±0.34) was also highly significant (p<0.001) but comparable. The above findings conclude that the anti-hyperglycaemic effectiveness of *terminalia arjuna* bark is comparable to the novel and effective drug Sitagliptin. In this study, the safety profile was assessed by monitoring the LFT and recording the adverse events that occurred after the start of treatment. None of the patients in any group had derangement of liver function tests and results values of SGOT, SGPT and ALP were comparable and non-significant (p > 0.05) at every follow-up visit. 2/30 patients had complaints of headache and 1/30 patients had nasopharyngitis in group SGM while none of the patients had any side effects in group TAM. Though statistically analyzing, this variation was non-significant (p > 0.05).

The present study findings are in-concordance with the findings of Ragavan *et al.* who stated [14] that oral administration of 250mg/kg and 500mg/kg of *T.arjuna* bark extract led to a significant reduction of blood glucose levels after 30 days in induced diabetic rats. They also found that the activity of certain enzymes' hexokinase, phosphoglucoisomerase, glucose-6-phosphatase, aldolase and fructose-1, 6-diphosphatase was also brought back to normal at a significant value after administration of *T.arjuna* in diabetic rats. Moreover, there was no detection of hypoglycaemia when *T.arjuna* was administered to the control group. Similarly, in our study, none of the patients had any incidence of hypoglycemia during the course of treatment. Another experimental study by Khan *et al.* [15] on high-fat diet and streptozocin induced type-2 diabetic rats, also stated an effective reduction in hyperglycemia, along with a reduction of hyperlipidaemia and oxidative stress related to the risk of diabetes and concluding the therapeutic value of *T.arjuna* in DM type 2. Further Morshed *et al.* [16] in an experimental study confirmed the significant enhancement in oral glucose tolerance and reduction in FBS level in type-2 diabetic rats after administration with ethanolic bark extract of *T.arjuna*, although this hypoglycaemic effect was gradual in nature, which is again consistent with the present study finding. Similarly, Mohanty *et al.* [13] and Borde *et al.* [17] confirmed the restoration of increased blood glucose levels as well as a reduction in HbA1c after the administration of *terminalia arjuna* in experimental diabetic rats, which was statistically significant and in concordance with the present study. Although they found the efficacy of Vildagliptin superior to *terminalia arjuna* as an anti-hyperglycemic agent, this finding is in contrast to our finding, where we found Sitagliptin and *T.arjuna* equally efficacious as anti-hyperglycemic agents. This difference may be due to their short study duration of 5 weeks as compare to present study of 12 weeks duration. Another experimental study by Biswas *et al.* 18] showed a highly significant (p<0.001) and dose-dependent decrease in blood sugar level with the administration of an extract of *T.arjuna* in streptozocin-induced diabetic rats. They also confirmed the superior efficacy of *T.arjuna* as compared to the reference drug glibenclamide as an anti-hyperglycaemic agent. Their liver function tests showed restoration of SGPT, SGOT, ALP levels to normal with *T.arjuna* administration. This finding is in concordance with ours. Further in this regard, a study done by Sawant *et al.* [19] in 40 newly diagnosed cases of T2DM with a dose of 6 gm daily for 3 months concluded an efficient reduction (p<0.05) in FBS level and HbA1c level with percentage relief of 18.69% and 14.75%, respectively. The results are in-concordance with the results of present study in regard to anti-hyperglycemic property of *T.arjuna*. Their study has a limitation as it was a single group study, without comparison group, thus decreasing the role in calculating comparative effectiveness, while our study has a comparison group.

## Conclusion:

The hypoglycemic property of potential medicinal plant- *terminalia arjuna* is described through various experimental studies; indeed literature has study, describing it to be a natural DPP- 4 inhibitor, which further draws attention of researchers towards it as potential anti-diabetic drug. Our present study with two different medical interventions, one with herbal-natural DPP-4 inhibitor *terminalia arjuna* bark and other with novel drug, Sitagliptin, a synthetic DPP-4 inhibitor demonstrated significant reduction in FBS and HbA1c in all enrolled patients, concluding that bark of *T.arjuna* possesses an efficient anti-hyperglycaemic property, although its efficacy was comparable to the efficacy of Sitagliptin but *T.arjuna* has a slight winning edge over the Sitagliptin, because no noticeable side effect was recorded in the patients. A longer study on a greater number of patients may be further helpful, leading to more conclusive opinion.

## Strength of study:

For all I know, the present study is a first clinical, two-group study design conducted to assess and analyze the anti-hyperglycemic potentiality of *terminalia arjuna* bark extract, with comparison with one of the most popular and novel anti-diabetic drug Sitagliptin.

## Limitation of study:

This study is conducted on limited number of patients for an average duration of 3months and component analysis of *T.arjuna* has not been performed.

## Source of support:

none

## Author contributions:

Conceptualization, Ved Prakash, Kiran Rajendra Giri, Ashish Goel; Data curation, Ved Prakash, Nidhi Goel; Formal analysis, Nidhi Goel and Kiran Rajendra Giri; Investigation, Nidhi Goel and Ved Prakash; Methodology, Ved Prakash and Ashish Goel; Writing - original draft, Ved prakash; Writing - review & editing, Ved Prakash, Kiran Giri and Nidhi Goel.

## Institutional review board statement:

Protocol approval as per HIMS/IRB/2020-21/253 from Hind Institute of Medical sciences, Barabanki, India, website: www.himsup.com

## Figures and Tables

**Table 1:** Demographic details and baseline characteristic comparison

**Characteristics**				**gpTAM (n=30)**	**gpSGM (n=30)**	**p-value/sig**
Mean age (years)				51.93±7.75	52.27±7.45	
	Male			12/30 (40%)	14/30 (46.7%)	
Gender	Female			18/30 (60%)	16/30 (53.3%)	
Chronicity of DM type-2 (years)				3.78±1.22	3.45±0.98	
			present	4/30 (13.3%)	5/30 (16.7%)	
	CHD		absent	26/30 (86.7%)	25/30 (83.3%)	
Past history			Present	14/30 (46.7%)	16/30 (53.3%)	
	HTN		absent	16/30 (53.3%)	14/30 (46.7%)	
			present	9/30 (30%)	7/30 (23.3%)	
	Alcohol		Absent	21/30 (70%)	23/30 (76.7%)	
			Present	8/30 (26.7%)	10/30 (33.3%)	
Personal history	Smoking		Absent	22/30 (73.3%)	20/30 (66.7%)	
			Present	17/30 (56.7%)	19/30 (63.3%)	>0.05/ NS
	Sedentary		Absent	13/30 (43.3%)	11/30 (36.7%)	
Baseline FBS				185.20±12.16	186.60±11.67	
Baseline HbA1c				7.44±0.47	7.53±0.51	
		SGPT		25.60±4.66	28.70±3.84	
Baseline Liver function test		SGOT		26.30±4.02	29.83±4.42	
		ALP		70.07±11.15	75.33±8.83	

**Table 2:** Mean change in FBS and HbA1c values of gpTAM at follow up visits (intragroup comparison)

**Parameter**	**Visits**	**Mean value**	**Mean change value**	**Standard error.M**	**t**	**Df**	**p-value**	**Sig.**
FBS	Baseline (start)	185.20±12.16	-	-	-	-		
	4weeks	162.62±13.09	22.57±9.28	1.69	13.32	29		
	8 weeks	143.07±10.33	42.13±8.95	1.64	25.78	29	<0.001	HS
	12 weeks	124.03±7.95	61.17±10.22	1.87	32.79	29		
HbA1c	Baseline	7.44±0.47	-	-	-	-		
	12 weeks	6.59±0.32	0.89±0.29	0.54	15.57	29	<0.001	HS

**Table 3:** FBS and HbA1c values of gpSGM at follow up visits (intragroup comparison)

**Parameter**	**Visits**	**Mean value**	**Mean change value**	**Standard error.M**	t	**df**	**P-value**	**Sig.**
FBS	Baseline (start)	186.60±11.67	-	-	-			
	4weeks	157.10±12.07	29.50±8.86	1.62	13.32	29	<0.001	HS
	8 weeks	140.57±11.19	46.03±9.73	1.81	25.38			
	12 weeks	123.80±7.56	62.80±9.06	1.65	37.96			
HbA1c	Baseline	7.53±0.51	-	-	-			
	12 weeks	6.63±0.29	0.90±0.34	0.61	14.13	29	<0.001	HS

**Table 4:** Comparing FBS & HbA1c values gpTAM vs gpSGM at follow up visits (intergroup comparison)

**Parameter**	**visits**	**Group**	**Mean±SD**	**Mean change difference**	**SE mean**	t	**df**	**p**	**Sig**
FBS	Baseline	TAM	185.20±12.16	-1.4	3.08	-0.46		0.65	
	(start)	SGM	186.60±11.67						
	4weeks	TAM	162.62±13.09	5.53	3.25	1.7		0.09	
		SGM	157.10±12.07						
	8 weeks	TAM	143.07±10.33	2.5	2.78	0.89		0.37	
		SGM	140.57±11.19				58		NS
	12 weeks	TAM	124.03±7.95	0.23	2.03	0.12		0.9	
		SGM	123.80±7.56						
HbA1c	Baseline	TAM	7.44±0.47	-0.09	0.13	-0.737		0.46	
	(start)	SGM	7.53±0.51						
	12 weeks	TAM	6.59±0.32	-0.04	0.08	-0.417	58	0.68	NS
		SGM	6.63±0.29						

**Table 5:** Intergroup comparison of LFT gpTAM vs gpSGM at follow up visits

**Parameter**	**visits**	**Group**	**Mean value**	**Mean change difference**	**Standard error.M**	**t**	**df**	**p-value**	**Sig.**
SGPT	Baseline	TAM	25.60±4.66	-3.1	1.1	-2.81	58	>0.05	NS
	(start)	SGM	28.70±3.84						
	4weeks	TAM	23.47±3.10	-2.47	1.03	-2.41			
		SGM	25.93±3.95						
	8 weeks	TAM	22.10±3.41	-1.73	0.94	-1.85			
		SGM	23.83±3.84						
	12 weeks	TAM	19.60±3.23	-1.37	0.85	-1.61			
		SGM	20.90±3.32						
SGOT	Baseline	TAM	26.30±4.02	-3.53	1.09	-3.24	58	>0.05	NS
		SGM	29.83±4.42						
	4weeks	TAM	24.93±3.52	-2.63	1.03	-2.55			
		SGM	27.57±4.42						
	8 weeks	TAM	23.03±3.24	-2.57	1	-2.57			
		SGM	25.60±4.42						
	12 weeks	TAM	22.07±2.79	-1.83	0.98	-1.87			
		SGM	23.90±4.60						
ALP	Baseline	TAM	70.07±11.15	-5.27	2.59	-2.03	58	>0.05	NS
		SGM	75.33±8.83						
	4weeks	TAM	65.70±8.99	-3.2	2.33	-1.38			
		SGM	68.90±9.03						
	8 weeks	TAM	60.60±7.80	-3.77	2.14	-1.76			
		SGM	64.37±8.75						
	12 weeks	TAM	56.07±7.56	-3.97	2.03	-1.95			
		SGM	60.03±8.19						
